# Associations of skeletal muscle volume and density with cardiovascular events in maintenance hemodialysis: a cross-sectional study

**DOI:** 10.1186/s12882-025-04591-5

**Published:** 2025-11-26

**Authors:** Zhaoting Li, Pingping Ju, Yu Zhu, Wenjie Yu, Jing He, Chunbo Zou

**Affiliations:** 1 Department of Nephrology, Taicang Loujiang New City Hospital (Ruijin Hospital Taicang Branch), Jiangsu, 215400 China; 2 Department of Nephrology, The Affiliated Taizhou People’s Hospital of Nanjing Medical University, Taizhou School of Clinical Medicine, Nanjing Medical University, Jiangsu, 225300 China; 3 Department of Nephrology, Sinopharm Tongmei General Hospital, Datong, 037003 China; 4 Department of Nephrology, The Affiliated Xuancheng Hospital of Wannan Medical College, Xuancheng, 242000 China; 5 Department of Nephrology, Suzhou Kowloon Hospital, Shanghai Jiao Tong University School of Medicine, Jiangsu, 215028 China

**Keywords:** Maintenance hemodialysis, Cardiovascular events, Skeletal muscle volume, Muscle density, Risk prediction

## Abstract

**Background:**

Cardiovascular events (CVEs) represent the leading cause of mortality in patients undergoing maintenance hemodialysis (MHD), particularly among older individuals with comorbid hypertension. While muscle volume and muscle density have emerged as promising markers associated with cardiovascular risk, their independent and synergistic roles in MHD patients remain insufficiently explored.

**Methods:**

In this cross-sectional study, a total of 391MHD patients from Taizhou People’s Hospital were enrolled. Skeletal muscle volume and density were quantified using axial computed tomography (CT) scans at the level of the first lumbar vertebra (L1), with image analysis performed via 3D Slicer software. Logistic regression analysis was applied to evaluate the associations between muscle-related parameters, age, hypertension, and CVEs. Model performance was assessed using receiver operating characteristic (ROC) curves and calibration plots.

**Results:**

Patients who experienced CVEs exhibited significantly lower skeletal muscle volume and density, along with higher prevalence of hypertension and diabetes (all *P* < 0.05). In multivariable logistic regression, both muscle volume [odds ratio (OR) per 1 SD = 0.60, *P* < 0.01] and muscle density (OR per 1 SD = 0.65, *P* = 0.016) were independently associated with reduced risk of CVEs. The model showed good discrimination [area under the curve (AUC) ≈ 0.82] and acceptable calibration.

**Conclusion:**

Skeletal muscle health—particularly higher muscle density—was associated with lower odds of CVEs in MHD patients. These findings support incorporating muscle quality metrics (e.g., density) into cardiovascular risk assessment, especially in older and hypertensive subgroups.

**Supplementary Information:**

The online version contains supplementary material available at 10.1186/s12882-025-04591-5.

## Introduction

Cardiovascular events (CVEs) remain a leading cause of morbidity and mortality worldwide, particularly among older individuals with hypertension, where the risk of adverse outcomes is significantly elevated [1]. Patients undergoing maintenance hemodialysis (MHD) face an even greater cardiovascular burden due to chronic hemodynamic instability, metabolic disturbances, and systemic inflammation [2]. In recent years, growing attention has been directed toward muscle health—specifically muscle volume and muscle density—as potential predictors of the risk of CVEs. Accumulating evidence suggests that loss of skeletal muscle mass in MHD patients is closely linked to an increased incidence of major adverse cardiovascular events, potentially mediated by disrupted metabolism, vascular calcification, and chronic inflammation [1]. Sheng et al. (2023) demonstrated that reduced skeletal muscle density (SMD) at the lumbar spine independently predicted both all-cause and cardiovascular mortality in incident dialysis patients [3]. Likewise, Yajima et al. (2022) found that lower psoas muscle index (PMI) and psoas muscle density (PMD) were significantly associated with higher all-cause mortality in MHD patients [4].

While muscle volume reflects total muscle mass, muscle density captures tissue quality, particularly the degree of fat infiltration. Both metrics serve as complementary indicators of muscle health, and reductions in either have been associated with vascular calcification, arteriovenous access failure, and elevated risk of CVEs [5]. For example, Larsen et al. (2023) reported that higher muscle density was inversely correlated with coronary heart disease, whereas muscle volume alone was insufficient to predict cardiovascular protection [6]. Furthermore, sarcopenia has been linked to increased cardiovascular and infection-related mortality in MHD populations [7]. Importantly, the cardioprotective role of muscle health may be attenuated in older MHD patients with comorbid hypertension. Prior studies have shown that sarcopenia is independently associated with hypertension in older adults, possibly via mechanisms involving metabolic dysfunction and impaired vascular tone [8]. Low skeletal muscle mass is also correlated with higher cardiovascular risk profiles—including elevated blood pressure, insulin resistance, and arterial stiffness—which are particularly prevalent in long-term dialysis patients [9]. Consequently, these observations suggest that, in older hypertensive individuals, muscle volume alone may be insufficient to evaluate cardiovascular risk, and greater emphasis should be placed on muscle quality metrics (e.g., density). Sato et al. (2024) underscored the role of chronic inflammation and vascular stiffness as key mechanisms mediating the diminished cardioprotective effects of muscle mass in this setting. Similarly, Yajima et al. (2025) highlighted the predictive value of PMI for adverse cardiovascular outcomes, especially in older MHD patients [10, 11].

Despite growing interest, systematic research evaluating the independent and synergistic effects of muscle volume, muscle density, age, and hypertension on the risk of CVEs remains scarce. To address this gap, the present study investigated patients undergoing MHD at the Hemodialysis Center of Taizhou People’s Hospital. Using 3D Slicer software, muscle volume and density were precisely measured from axial computed tomography (CT) images at the first lumbar vertebra (L1) level. Through comprehensive analysis of the independent and interactive influences of muscle parameters, age, and hypertension on the risk of CVEs, this study aims to provide supportive evidence to guide personalized cardiovascular risk stratification and inform targeted prevention and management strategies in clinical practice.

Given the cross-sectional design, our analyses focus on associations rather than causality.

## Study population

This cross-sectional, retrospective study enrolled patients receiving maintenance hemodialysis (MHD) at Taizhou People’s Hospital between January 2023 and January 2024. Inclusion criteria were: age ≥ 18 years; dialysis duration ≥ 3 months; availability of L1 CT-based muscle measurements; documented CVEs status; and complete clinical/laboratory data. Exclusion criteria were: dialysis duration < 3 months; >20% missing key variables; or acute illness (e.g., myocardial infarction, acute kidney injury) at enrollment. Because some patients had experienced CVEs before enrollment, reverse causation was considered when interpreting results.

### Data collection and measurement

Baseline demographic and clinical data were collected, including age, sex, blood pressure, diabetes, hypertension, use of lipid-lowering or antiplatelet drugs, and lifestyle history. CVEs were defined as myocardial infarction, heart failure, stroke, or cardiovascular death, adjudicated by two nephrologists.

Skeletal muscle volume was quantified using 3D Slicer software. At the level of L1, skeletal muscle was manually segmented on axial CT images, and the segmented regions were reconstructed into a three-dimensional model. Muscle volume was then calculated automatically by the software based on voxel counts and slice thickness, providing a volumetric estimate rather than a single cross-sectional area. Skeletal muscle density was defined as the mean CT attenuation in Hounsfield units (HU), reflecting tissue composition and fat infiltration. Although the third lumbar vertebra (L3) is more widely validated, prior studies indicate reasonable correlations between L1- and L3-derived measures [3, 12]; thus, L1 was used for pragmatic reasons in this single-center cohort. This choice was acknowledged as a limitation.

Additional details of CT acquisition, segmentation reliability, and laboratory assays are provided in the Supplementary Methods.

#### Laboratory indicators

Pre-dialysis venous blood was sampled for biochemical tests, including serum creatinine, urea, electrolytes, lipid profile, apolipoprotein B (ApoB), hemoglobin, B-type natriuretic peptide (BNP), parathyroid hormone (PTH), hemoglobin A1c (HbA1c), C-reactive protein (CRP), following standardized laboratory procedures.

### Statistical analysis

Continuous variables were expressed as mean ± standard deviation or median (IQR), categorical variables as counts and percentages. Group comparisons used the t-test, Mann-Whitney U test, or χ² test as appropriate. Logistic regression was used to evaluate associations between muscle parameters and CVEs. Model discrimination was assessed by receiver operating characteristic (ROC) curves and calibration plots. Comparisons of AUCs between models were descriptive; no formal statistical test (e.g., DeLong) was performed. Primary analyses used complete-case data; multiple imputation (m = 5) was used only for sensitivity analyses (LASSO and the forced-covariate models). In multivariable models, continuous predictors were z-score standardized and ORs are reported per 1 SD; in univariate models, ORs are reported per clinically interpretable increments as specified in Table 2.

### Sensitivity analyses consisted of two approaches

Least absolute shrinkage and selection operator (LASSO) regression: Performed across five imputed datasets, with coefficient path and cross-validation plots shown in Supplementary Figures S1 and S2.

Logistic regression with forced inclusion of key covariates: age, diabetes, and albumin were forced into the baseline model; results are provided in Supplementary Table S1. A comparison across modeling strategies (primary model, LASSO, and forced-inclusion model) is presented in Supplementary Table S2.

All sensitivity findings were directionally consistent with the primary analyses.

## Results

### Baseline characteristics

A total of 391 patients receiving MHD were included in the analysis, comprising 253 without CVEs and 138 with CVEs. Baseline comparisons revealed significant differences in age, muscle volume, muscle density, blood pressure, lipid profiles, and inflammatory markers between the two groups (all *P* < 0.05; Table 1). Specifically, patients in the CVEs group were significantly older and had elevated blood pressure, HbA1c, and BNP levels. In contrast, their muscle volume and muscle density were significantly lower. These findings suggest that advanced age, hypertension, impaired glucose metabolism, systemic inflammation, and reduced muscle quality metrics (e.g., density) are associated with increased risk of CVEs in MHD patients.

**Table 1:** Baseline characteristics of patients according to the presence or absence of cardiovascular events (CVEs)

Variable	Without CVEs	With CVEs	*P*-value
Hypertension, n (%)	196 (77.5%)	125 (90.6%)	< 0.010
Diabetes, n (%)	76 (30.0%)	62 (44.9%)	< 0.010
Lipid-lowering medication, n (%)	47 (18.6%)	54 (39.1%)	< 0.010
Antiplatelet medication, n (%)	67 (26.5%)	65 (47.1%)	< 0.010
Albumin, g/L	36.0 (31.3, 39.2)	34.1 (29.7, 36.8)	< 0.010
B-type natriuretic peptide, pg/mL	232.45 (75.54, 778.66)	784.70 (293.99, 1619.99)	< 0.010
Parathyroid hormone, pg/mL	268.9 (149.3, 437.0)	199.6 (114.7, 318.9)	< 0.010
Serum creatinine, µmol/L	843.7 (669.9, 1021.7)	711.1 (548.9, 894.3)	< 0.010
Total cholesterol, mmol/L	3.7 (3.0, 4.4)	3.3 (2.7, 4.0)	< 0.010
Neutrophil percentage, %	71.8 (64.8, 78.7)	75.0 (67.9, 81.5)	0.016
LDL-C, mmol/L	2.3 (1.8, 2.8)	2.0 (1.5, 2.5)	< 0.010
Age, years	54.0 (45.0, 65.0)	62.5 (55.0, 72.0)	< 0.010
Inorganic phosphate, mmol/L	1.9 (1.5, 2.4)	1.7 (1.3, 2.3)	0.024
Lymphocyte percentage, %	17.9 (12.6, 23.0)	14.9 (8.8, 20.9)	< 0.010
Muscle volume, mm^3^	52380.9 (43098.2, 63668.1)	49107.9 (39980.8, 60344.1)	0.044
Muscle density, HU	33.4 ± 7.1	28.5 ± 8.1	< 0.010
Hemoglobin, g/L	93.0 (76.0, 111.0)	86.5 (72.2, 104.8)	0.027
Apolipoprotein B, g/L	0.7 (0.5, 0.9)	0.6 (0.5, 0.8)	0.022
Hemoglobin A1c, %	5.7 (5.2, 6.3)	5.9 (5.4, 6.8)	0.039

Note: Data are presented as mean ± SD or median (IQR) for continuous variables and n (%) for categorical variables. Two-sided P values compare patients with vs. without CVEs. Normality was assessed with the Shapiro–Wilk test and homogeneity of variances with Levene’s test; independent-samples *t* tests were used when both assumptions were met, otherwise the Mann–Whitney U test was applied. For categorical variables, the χ² test was used (Fisher’s exact test when expected cell counts < 5)

**Abbreviations**: CVEs, cardiovascular events; LDL-C, low-density lipoprotein cholesterol; SD, standard deviation; IQR, interquartile range

### Univariate analysis

Univariate comparisons showed that patients with CVEs had lower muscle volume and density, and higher rates or levels of hypertension, HbA1c, and BNP (all *P* < 0.05; Table 2). Among these variables, the between-group contrast for muscle density was among the more pronounced by P-value rank, suggesting a stronger inverse association signal at the univariate level.

**Table 2:** Univariate analysis of factors associated with the occurrence of cardiovascular events (CVEs)

Variable	OR (95%CI)	*P*-value
Hypertension	2.78 (1.47–5.26)	< 0.010
Diabetes	1.89 (1.23–2.94)	< 0.010
Lipid-lowering medication	2.78 (1.75–4.55)	< 0.010
Antiplatelet medication	2.44 (1.59–3.85)	< 0.010
Albumin, g/L	0.93 (0.90–0.97)	< 0.010
B-type natriuretic peptide, pg/mL	1.03 (1.01–1.05)	< 0.010
Parathyroid hormone, pg/mL	0.98 (0.99–1.00)	< 0.010
Serum creatinine, µmol/L	0.98 (0.97–0.99)	< 0.010
Total cholesterol, mmol/L	0.74 (0.60–0.91)	< 0.010
Neutrophil percentage, %	1.02 (1.00–1.04)	0.049
LDL-C, mmol/L	0.60 (0.45–0.81)	< 0.010
Age, years	1.05 (1.03–1.07)	< 0.010
Inorganic phosphate, mmol/L	0.68 (0.50–0.93)	0.014
Lymphocyte percentage, %	0.96 (0.94–0.99)	< 0.010
Muscle volume, mm^3^	0.98 (0.97–0.99)	0.012
Muscle density, HU	0.92 (0.89–0.94)	< 0.010
Hemoglobin, g/L	0.99 (0.98–1.00)	0.043
Apolipoprotein B, g/L	0.25 (0.09–0.73)	< 0.010
Hemoglobin A1c, %	1.19 (1.00–1.40)	0.039

Note: Univariate logistic regression with CVE as the binary outcome. For continuous predictors, ORs are per 1 unit increase (as shown in the left column) except: Serum Creatinine per 10 µmol/L; Parathyroid Hormone per 10 pg/mL; B-type natriuretic peptide per 100 pg/mL; Muscle Volume per 1000 mm³. Binary categorical predictors are coded 1 = Yes and 0 = No; ORs compare Yes vs. No (reference). Two-sided P values; complete-case analysis

**Abbreviations**: OR, odds ratio; CI, confidence interval; CVEs, cardiovascular events

### Multivariate analysis

**Fig. 1:**
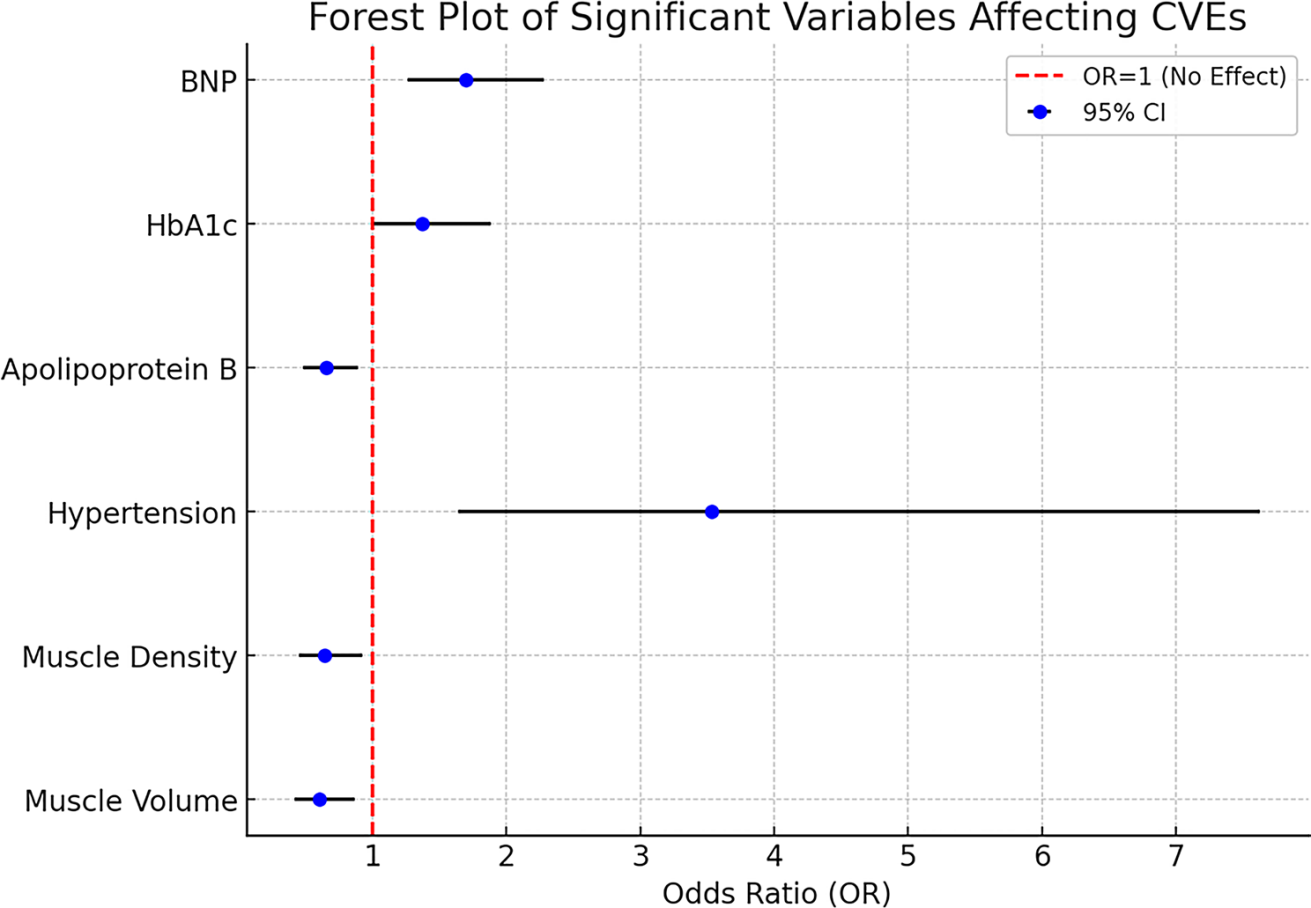
Forest plot of multivariable **odds ratios** for factors associated with CVEs

After adjusting for hypertension, HbA1c, ApoB, and BNP (primary model), both muscle volume (OR per 1 SD = 0.60, *P* < 0.01) and muscle density (OR per 1 SD = 0.65, *P* = 0.016) remained independently associated with lower odds of CVEs (Table 3; Fig. 1). Hypertension (OR = 3.53, *P* < 0.01) and HbA1c (OR = 1.37, *P* = 0.048) persisted as significant risk factors, whereas ApoB (OR = 0.66, *P* < 0.01) demonstrated a protective association. These results underscore the independent prognostic value of muscle-related parameters, even after controlling for conventional cardiovascular risk factors.

**Table 3:** Multivariable logistic regression analysis of factors associated with cardiovascular events (CVEs)

Variable	OR (95%CI)	*P*-value
Muscle volume, mm^3^	0.60 (0.42, 0.86)	< 0.010
Muscle density, HU	0.65 (0.45, 0.93)	0.016
Hypertension, n (%)	3.53 (1.64, 7.63)	< 0.010
Apolipoprotein B, g/L	0.66 (0.48, 0.90)	< 0.010
Hemoglobin A1c, %	1.37 ( 1.00, 1.88)	0.048
B-type natriuretic peptide, pg/mL	1.70 (1.26, 2.28)	< 0.010

Note: ORs are per 1 standard deviation (SD) increase. Binary categorical predictors are coded 1 = Yes and 0 = No; ORs compare Yes vs. No (reference). Two-sided P values; complete-case analysis. Adjustment set: muscle volume, muscle density, hypertension, apolipoprotein B, hemoglobin A1c, and BNP

**Fig. 2:**
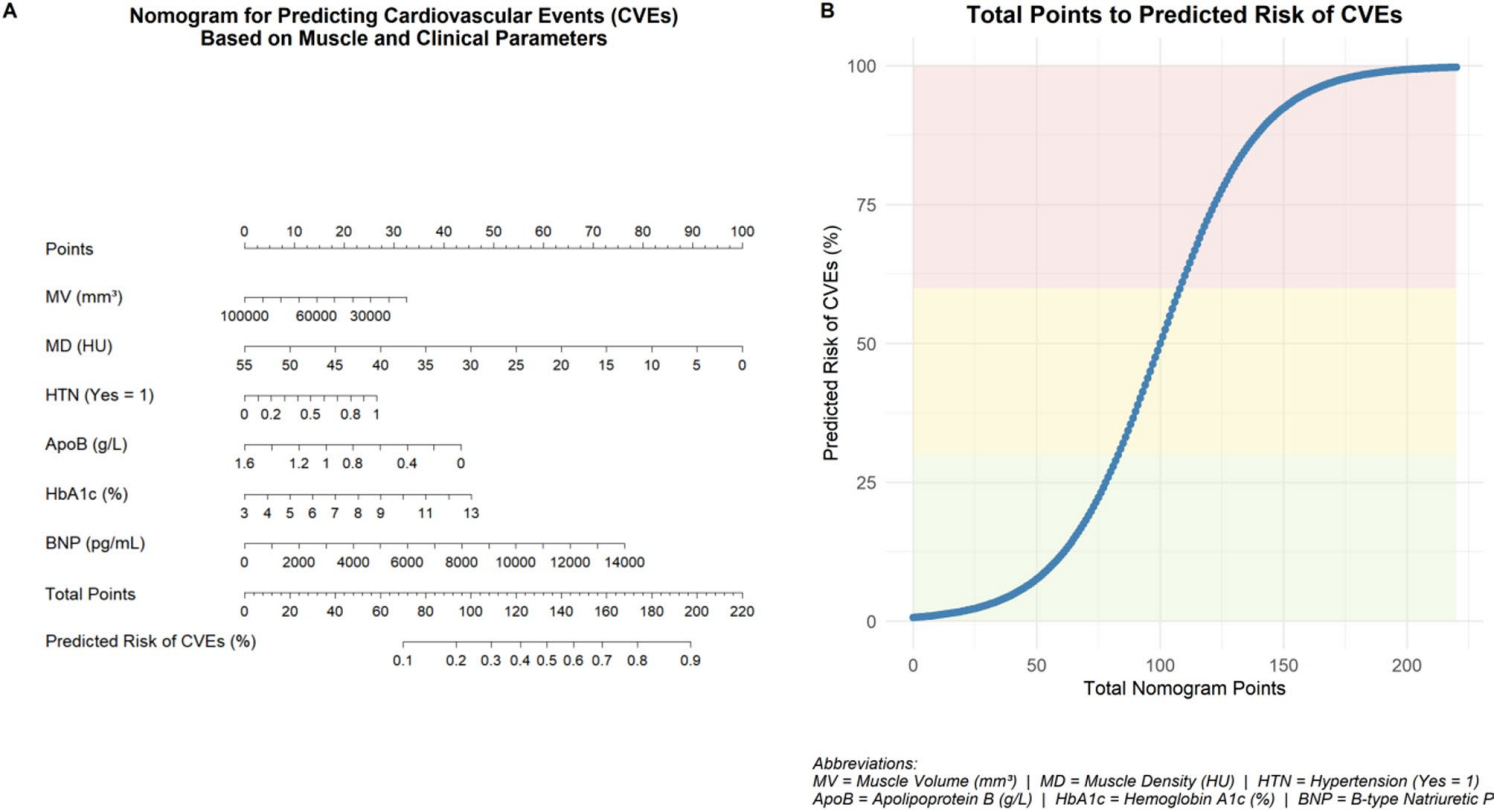
Nomogram and risk conversion curve for individualized CVE risk estimation. (**A**) Nomogram derived from the multivariable logistic model including muscle volume (MV), muscle density (MD), hypertension (HTN, Yes = 1), apolipoprotein B (ApoB), hemoglobin A1c (HbA1c), and B-type natriuretic peptide (BNP). How to use: (i) locate the patient’s value/level on each predictor axis and read the corresponding Points; (ii) sum all points to obtain Total Points; (iii) map Total Points to the bottom scale to obtain the predicted probability of CVE. This nomogram is presented as an illustrative representation of relative contributions in our cross-sectional model and is not intended for clinical decision-making. (**B**) Risk conversion curve translating Total Points to predicted probability of CVE

In a sensitivity model that additionally included age, diabetes, and albumin, muscle density, muscle volume, hypertension, and ApoB remained significant variables associated with CVEs, whereas HbA1c lost significance. These findings indicate that the primary results were largely consistent after adjustment for key confounders. Based on these predictors, a nomogram was constructed to facilitate individualized risk estimation for CVEs (Fig. 2).

### Nomogram and risk translation visualization

As shown in Fig. 2, a nomogram was constructed based on variables independently associated with CVEs to provide individualized risk estimates. Panel A illustrates the point contribution of each variable, while Panel B presents a risk conversion curve that translates total scores into predicted probabilities. The nomogram was provided as an **illustrative visualization** of relative contributions and was **not intended for clinical decision-making**.

### Model performance

**Fig. 3:**
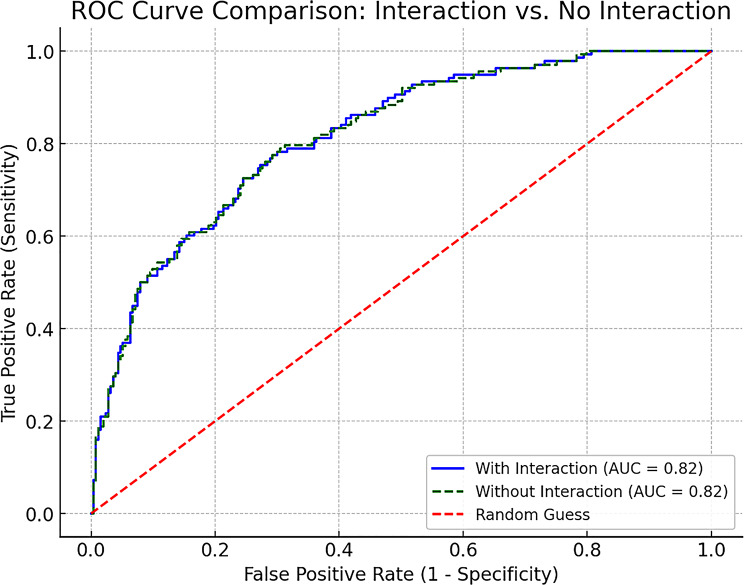
Receiver operating characteristic (ROC) curves for models with and without the interaction term in predicting CVEs. Both models showed similar discrimination (AUC ≈ 0.82)

Adding the interaction term yielded a negligible change in discrimination [area under the curve (AUC) = 0.8162 vs. 0.8161, **descriptive comparison only**, no DeLong test], which is unlikely to be clinically meaningful (Fig. 3). Given the marginal change in discrimination when adding the interaction term, we interpret this finding as hypothesis-generating: in older hypertensive patients, muscle mass alone may be insufficient for risk stratification and should be interpreted alongside density and comorbidity burden.

### Model calibration

**Fig. 4:**
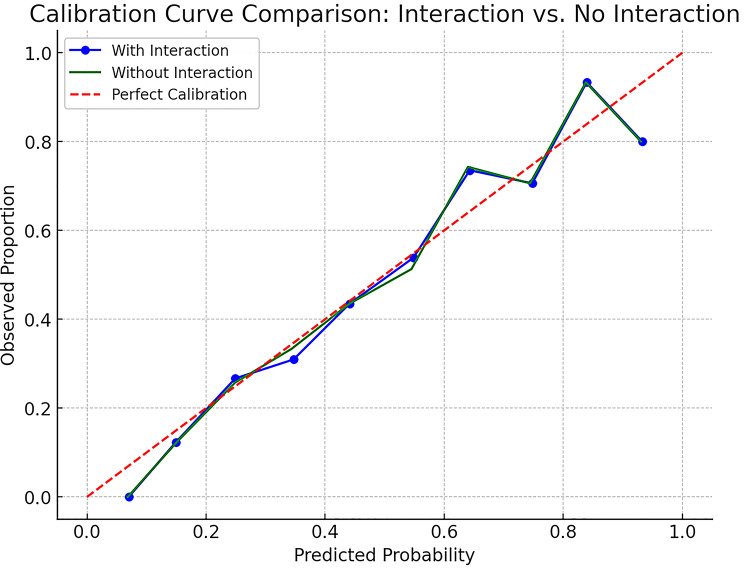
Calibration of the multivariable model. Apparent calibration was assessed in the derivation cohort (*n* = 391) by plotting observed proportions against predicted probabilities across risk groups with loess smoothing. The 45° diagonal denotes perfect calibration (predicted equals observed). Curves are shown for models with and without the interaction term; both exhibited good agreement with observed outcomes. No internal resampling was performed; calibration reflects apparent performance in the study sample

Calibration curves were generated to evaluate the agreement between predicted and observed event probabilities. As illustrated in Fig. 4, both models—with and without the interaction term—exhibited good calibration, with prediction curves closely aligning with the ideal 45-degree reference line. These findings indicate that the predicted risks are consistent with actual outcomes, reinforcing the reliability and clinical applicability of the nomogram-based estimates. Calibration reflects apparent performance in the derivation cohort; no internal resampling was performed.

### Dose-response relationship between muscle parameters and cardiovascular risk

**Fig. 5:**
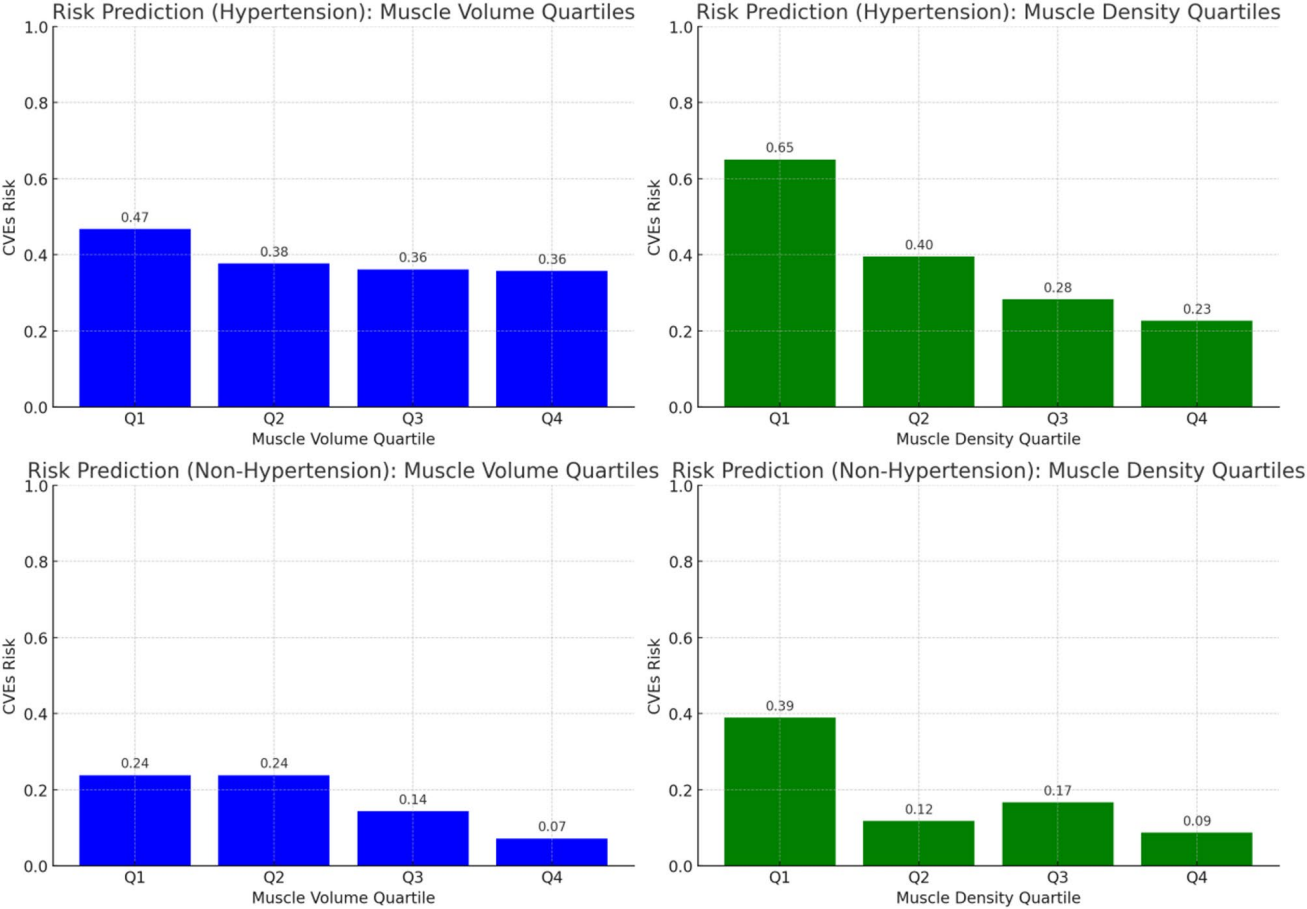
Dose-response relationships between muscle parameters (quartiles) and the risk of CVEs, stratified by hypertension status

**Fig. 6:**
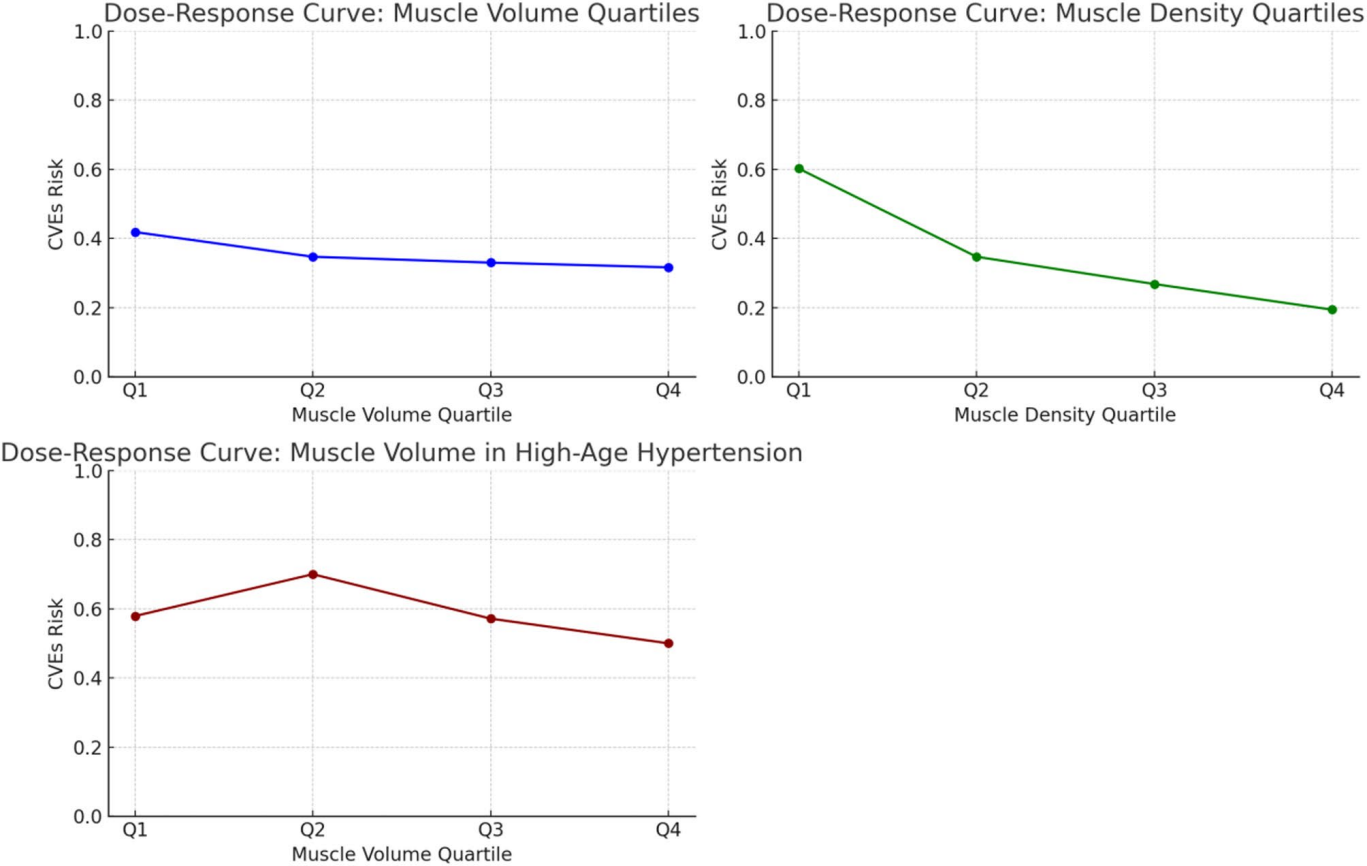
Dose-response relationships between muscle parameters and the risk of CVEs in older patients with hypertension

To comprehensively evaluate the relationship between muscle health and the risk of CVEs, both muscle volume and muscle density were stratified into quartiles (Q1-Q4). A clear dose-response trend was observed, with the risk of CVEs progressively decreasing from Q1 to Q4 (Figs. 5 and 6). Among these, the highest quartile of muscle density (Q4) demonstrated the strongest protective effect.

Further stratified analysis by hypertension status revealed variations in protective trends across subgroups. In hypertensive individuals, the association between muscle volume and the risk of CVEs showed a modest decline across quartiles (0.47, 0.38, 0.36, and 0.36 from Q1 to Q4), indicating a relatively weaker protective effect. In contrast, a more pronounced inverse association was observed among non-hypertensive individuals, where the risk of CVEs decreased from 0.24 in Q1 and Q2 to 0.14 in Q3 and 0.07 in Q4. These findings suggest that higher muscle volume may provide greater cardiovascular protection in low-risk populations without hypertension, while muscle density remains a stable and independent predictor of CVEs across all subgroups.

### Interaction effect analysis

**Fig. 7:**
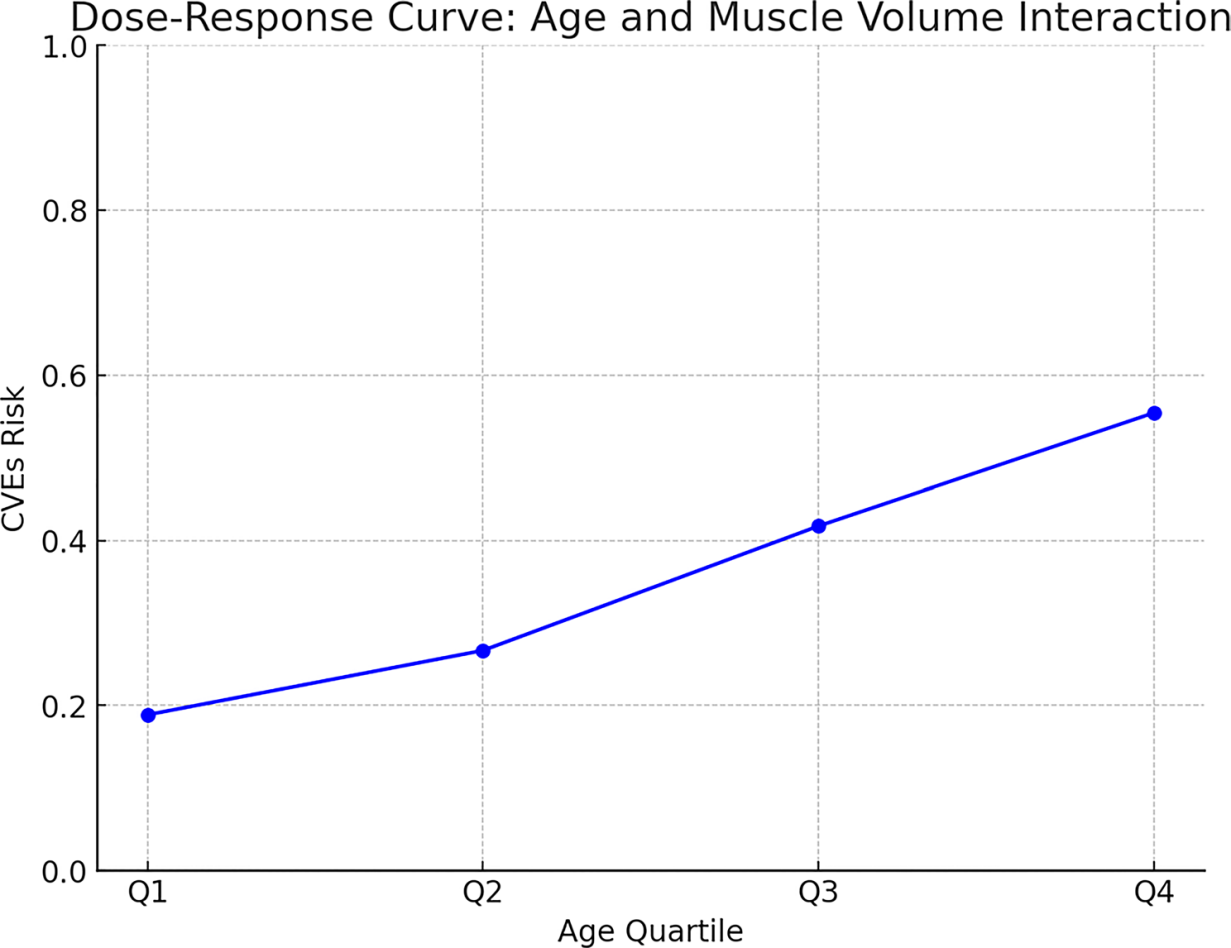
Interaction plot showing the effect modification of age and hypertension on the association between muscle volume and the risk of CVEs

To further investigate the interplay between muscle characteristics and clinical variables, an interaction term (age × muscle volume × hypertension) was introduced into the multivariable model and found to be statistically significant (*P* < 0.05). As shown in Fig. 7, the protective effect of muscle volume was substantially attenuated in older patients with hypertension, indicating that muscle mass alone may be insufficient to mitigate cardiovascular risk in this high-risk subgroup. These findings suggest that, in such populations, a more refined assessment incorporating muscle quality metrics (e.g., density) and composition may be necessary for accurate risk stratification.

Clinically, these findings imply that in older patients with hypertension, relying on muscle mass alone may be insufficient for risk stratification. A more comprehensive approach that incorporates muscle quality metrics (e.g., density) and concomitant cardiovascular comorbidities may better reflect residual risk.

## Discussion

CVEs remain the leading cause of mortality in patients undergoing MHD, with particularly high rates observed in older and hypertensive populations. In recent years, increasing attention has been directed toward muscle health as a modifiable factor in cardiovascular risk prediction. Among various indicators, muscle volume and muscle density have emerged as variables associated with lower CVE risk. However, given the distinct metabolic and inflammatory milieu in MHD patients, the applicability of these markers in this population requires further validation.

In this study, L1-level CT images were analyzed using 3D Slicer software to obtain accurate measurements of muscle volume and density in MHD patients. By correlating these measures with CVEs incidence, we observed that both muscle volume and muscle density were independently associated with lower odds of CVEs. Logistic regression analyses confirmed their discriminative association, while subgroup analyses highlighted differential effects based on age and hypertensive status. These findings support considering muscle health parameters in risk stratification for MHD patients; prospective validation is needed before clinical implementation.

Prior studies have shown that increasing muscle mass improves basal metabolic rate and insulin sensitivity, thereby reducing cardiometabolic burden [13]. Additionally, greater muscle volume is associated with enhanced blood pressure stability, potentially mitigating hypertensive damage [14]. Kim et al. reported a strong link between reduced muscle mass and higher risk of CVEs in MHD patients [15]. Similar findings from Visser et al. and Liu et al. confirmed that low muscle mass and low SMD were significantly associated with both cardiovascular and all-cause mortality in dialysis populations [16, 17]. Our findings align with this body of evidence and are consistent with lower odds of CVEs among patients with greater muscle volume, although causality cannot be inferred.

In addition to muscle quantity, this study highlights the critical role of muscle density, a muscle quality metric reflecting fat infiltration. Higher muscle density was independently associated with reduced risk of CVEs, even in high-risk subgroups. Previous studies have shown that low muscle density is associated with increased intramuscular fat accumulation, which promotes inflammation through upregulation of cytokines such as interleukin-6 (IL-6) and tumor necrosis factor-α (TNF-α), thereby accelerating atherosclerosis [18]. Furthermore, muscle density has been linked to metabolic syndrome and coronary artery disease, lending further support to its clinical relevance [19, 20]. Recent machine learning studies also identified imaging markers such as psoas muscle characteristics as predictors of CVEs in chronic kidney disease and dialysis populations [21]. Taken together, these findings support muscle density as a potentially clinically meaningful biomarker associated with CVEs.

### Subgroup analysis: older and hypertensive patients

In subgroup analyses, sarcopenic obesity (SO), defined by reduced skeletal muscle mass coexisting with increased adiposity, emerged as a significant risk factor for CVEs, particularly among older and hypertensive patients. This phenotype likely represents a convergence of metabolic derangement and vascular vulnerability. This dual burden impairs metabolic function and increases inflammatory load, amplifying cardiovascular vulnerability [20, 22]. The combination of reduced muscle reserve and visceral adiposity results in diminished metabolic buffering capacity and vascular stiffness, accelerating disease progression. In our sample, SO was associated with higher odds of adverse cardiovascular outcomes in MHD patients; residual confounding cannot be excluded [20].

### Hypertension and muscle loss: a converging risk pathway

Hypertension, a prevalent complication in MHD, may exacerbate muscle wasting through impaired perfusion and chronic inflammation [8]. In this cohort, patients with coexisting sarcopenia and hypertension had higher odds of CVEs. This suggests that muscle deterioration in the context of hypertension may further compromise vascular health via metabolic and endothelial dysfunction [8, 23]. Accordingly, simultaneous blood pressure control and muscle preservation should be prioritized in cardiovascular risk management, particularly in older MHD patients.

Skeletal muscle also plays a central role in glucose metabolism, serving as the body’s largest reservoir for glucose uptake. High-quality muscle tissue enhances insulin signaling and reduces insulin resistance, thereby improving metabolic syndrome profiles and reducing cardiovascular risk [1, 24]. These mechanistic insights support the clinical importance of maintaining muscle health in the comprehensive management of MHD patients.

### Muscle density, fat infiltration, and inflammation

Low muscle density often reflects intramuscular fat accumulation, which fosters localized inflammation and contributes to vascular injury. Adipose tissue-derived cytokines, including TNF-α and IL-6, promote oxidative stress and endothelial dysfunction, key drivers of atherosclerosis [13, 18]. Moreover, the pattern of fat distribution—particularly visceral versus subcutaneous fat—has been shown to differentially influence cardiovascular outcomes [25]. These findings underscore the need to address both muscle quality metrics (e.g., density), and adiposity in risk stratification and intervention strategies.

### Resistance training: a practical intervention

Resistance training has been shown to improve both muscle volume and density, offering a non-pharmacological approach to cardiovascular risk reduction [14, 26]. Enhanced muscle strength stabilizes blood pressure and mitigates cardiovascular stress [10]. Notably, even minimal-dose resistance exercise has been effective in improving muscle health among older MHD patients, offering a feasible and impactful strategy to reduce the dual burden of SO [5]. These results support the incorporation of structured exercise programs into routine care to improve outcomes in this vulnerable population.

### Clinical significance and future perspectives

This study is the first to utilize 3D Slicer software for precise quantification of muscle volume and muscle density in patients undergoing MHD, establishing both parameters as independently associated with lower odds of CVEs. These findings provide important evidence supporting the individualized management of high-risk subpopulations, particularly older patients with hypertension.

Among the two indicators, muscle density showed a more stable and consistent association, highlighting its potential for clinical application pending external validation. Incorporating muscle density into risk stratification may help identify higher-risk individuals; however, prospective studies are required. For those with low muscle density, individualized interventions—including resistance training and targeted nutritional support—could serve as effective strategies to reduce cardiovascular risk.

Given the dynamic nature of muscle quality metrics (e.g., density), longitudinal follow-up using 3D Slicer is warranted to assess changes in muscle density over time and evaluate its long-term association with cardiovascular outcomes. Such ongoing surveillance would not only validate the durability of muscle density’s protective effect but also contribute to evidence-based, personalized care strategies in MHD populations.

Clinically, a dual approach of enhancing muscle mass and reducing intramuscular fat infiltration may offer a feasible path to lowering cardiovascular risk.

To further strengthen our conclusions, we conducted a sensitivity analysis by incorporating age, diabetes, and albumin into the multivariable model, as suggested by reviewers. The results were consistent with the primary analysis, supporting the robustness of our findings. Nevertheless, the limited number of CVEs may reduce statistical power, and larger multicenter studies are warranted. Future research should further investigate the regional variation in muscle group contributions to cardiovascular protection and validate these findings across larger, multicenter cohorts [4, 27] to ensure generalizability and clinical translation.

#### Research limitations

This study has several limitations. First, its cross-sectional design precludes causal inference, and the observed associations should be interpreted accordingly. Second, reverse causation cannot be excluded, as some patients had prior CVEs and reduced muscle mass may be a consequence rather than a cause. Third, muscle measurements were obtained at the L1 level instead of the better validated L3, which may introduce variability. Fourth, the modest sample size, particularly the limited number of CVEs, may reduce power for subgroup and interaction analyses. Future large, multicenter prospective studies are warranted to validate these findings and clarify causal pathways.

## Conclusion

Both muscle volume and density were independently associated with lower odds of CVEs in MHD patients. Notably, muscle density showed a more consistent association, including among older and hypertensive subgroups. These findings underscore the associational value of muscle quality metrics for risk stratification in MHD and support their consideration in assessment frameworks, subject to external and longitudinal validation. Given the cross-sectional design, no causal inferences can be made.

## Supplementary Information

Below is the link to the electronic supplementary material.

Supplementary Material 1

## Data Availability

The datasets generated and/or analyzed during the current study are available from the corresponding author upon reasonable request.

## Abbreviations

MHD: Maintenance hemodialysis
CVEs: Cardiovascular events
CT: Computed tomography
L1/L3: First/third lumbar vertebra
HU: Hounsfield unit
ROC: Receiver operating characteristic
AUC: Area under the curve
IQR: Interquartile range
HbA1c: Hemoglobin A1c
ApoB: Apolipoprotein B
BNP: B-type natriuretic peptide
PTH: Parathyroid hormone
CRP: C-reactive protein
PMI: Psoas muscle index
PMD: Psoas muscle density
SMD: Skeletal muscle density
SO: Sarcopenic obesity
LASSO: Least absolute shrinkage and selection operator
LDL-C: Low-density lipoprotein cholesterol
