# Supplementary material for: Associations of skeletal muscle volume and density with cardiovascular events in maintenance hemodialysis: a cross-sectional study
Source: BMC Nephrol. 2025 Nov 26;26:673. doi: 10.1186/s12882-025-04591-5 (PMC12659376; doi:10.1186/s12882-025-04591-5)
Supplement: Supplementary file 1 — Supplementary Material 1 [file 12882_2025_4591_MOESM1_ESM.docx]

**Supplementary Methods**

**CT Acquisition and Image Analysis**

CT scans were acquired with standardized parameters (120 kVp, 250 mA, 5 mm slice thickness). At the L1 level, skeletal muscle was manually segmented using 3D Slicer (version 5.2.2). Segmented regions were reconstructed into a three-dimensional model, from which skeletal muscle volume was automatically calculated based on voxel counts and slice thickness. Muscle density was defined as the mean CT attenuation in Hounsfield units (HU), reflecting tissue composition and fat infiltration. Two radiologists independently performed the analyses, and inter-rater reliability was assessed using intraclass correlation coefficients (ICCs).

**Missing Data and Outliers**

Primary analyses used complete-case data; multiple imputation (m = 5) was used only for sensitivity analyses. Imputations assumed missing at random. Variables with >20% missingness were excluded. Outliers were identified using the interquartile range (IQR) method; no observations were removed solely on the basis of being an outlier.

**Multicollinearity**

Variance inflation factors (VIF) were used to evaluate collinearity. Variables with VIF >10 were excluded.

**Nomogram Construction**

Independent predictors from the multivariable logistic regression were incorporated into a nomogram to provide individualized cardiovascular risk estimates. Continuous predictors were z-score standardized (effects per 1 standard deviation), and total points were mapped to predicted probability as shown in Figure 3. The nomogram is provided as an illustrative visualization and is not intended for clinical decision-making.

**Sensitivity Analyses**

**Two sensitivity analyses were performed.**

(1) LASSO regression: conducted across five imputed datasets (m = 5) with 10-fold cross-validation using AUC as the metric. The coefficient path and cross-validation plots are shown in Supplementary Figures S1 and S2. The penalty was chosen at λ.1se (the largest λ within one standard error of the maximum mean AUC) to favor parsimony.

(2) Logistic regression with forced inclusion of key covariates: age, diabetes, albumin, and BNP were forced into the baseline model in addition to the original predictors. Results are provided in Supplementary Table S1. A comparison of variables identified across the three modeling strategies (primary model, LASSO at λ.1se, and forced-inclusion model) is summarized in Supplementary Table S2.

All sensitivity findings were directionally consistent with the primary analyses.


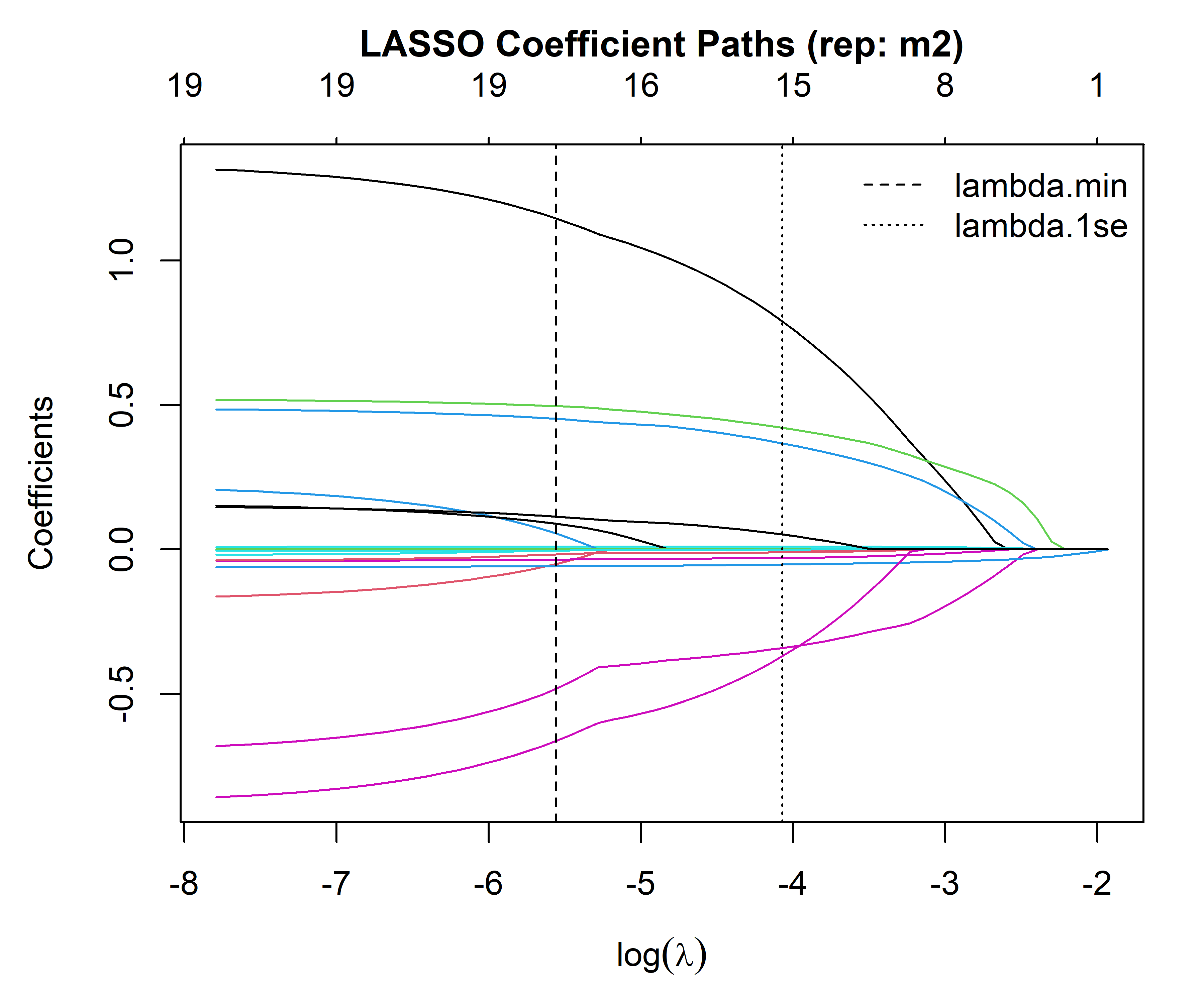


**Figure S1. LASSO coefficient paths (imputed dataset m2; sensitivity analysis).**
This figure illustrates the role of LASSO as a sensitivity analysis on multiply imputed data (m = 5), used to stabilize variable selection and assess robustness of the primary logistic model. Each colored curve represents the coefficient trajectory of a candidate predictor as the penalty parameter λ increases (x-axis, log scale): coefficients shrink toward zero with stronger penalization, and variables that cross zero are effectively excluded. Vertical dashed line marks λ.min (the λ yielding the highest mean AUC in 10-fold cross-validation), while the vertical dotted line marks λ.1se (the largest λ within one standard error of the maximum AUC). We adopted λ.1se to favor parsimony and minimize overfitting. Variables retained at λ.1se are summarized in Supplementary Table S2, and their directions were consistent with the primary multivariable model. (Panel shown for imputed dataset m2; patterns were similar across imputations.)


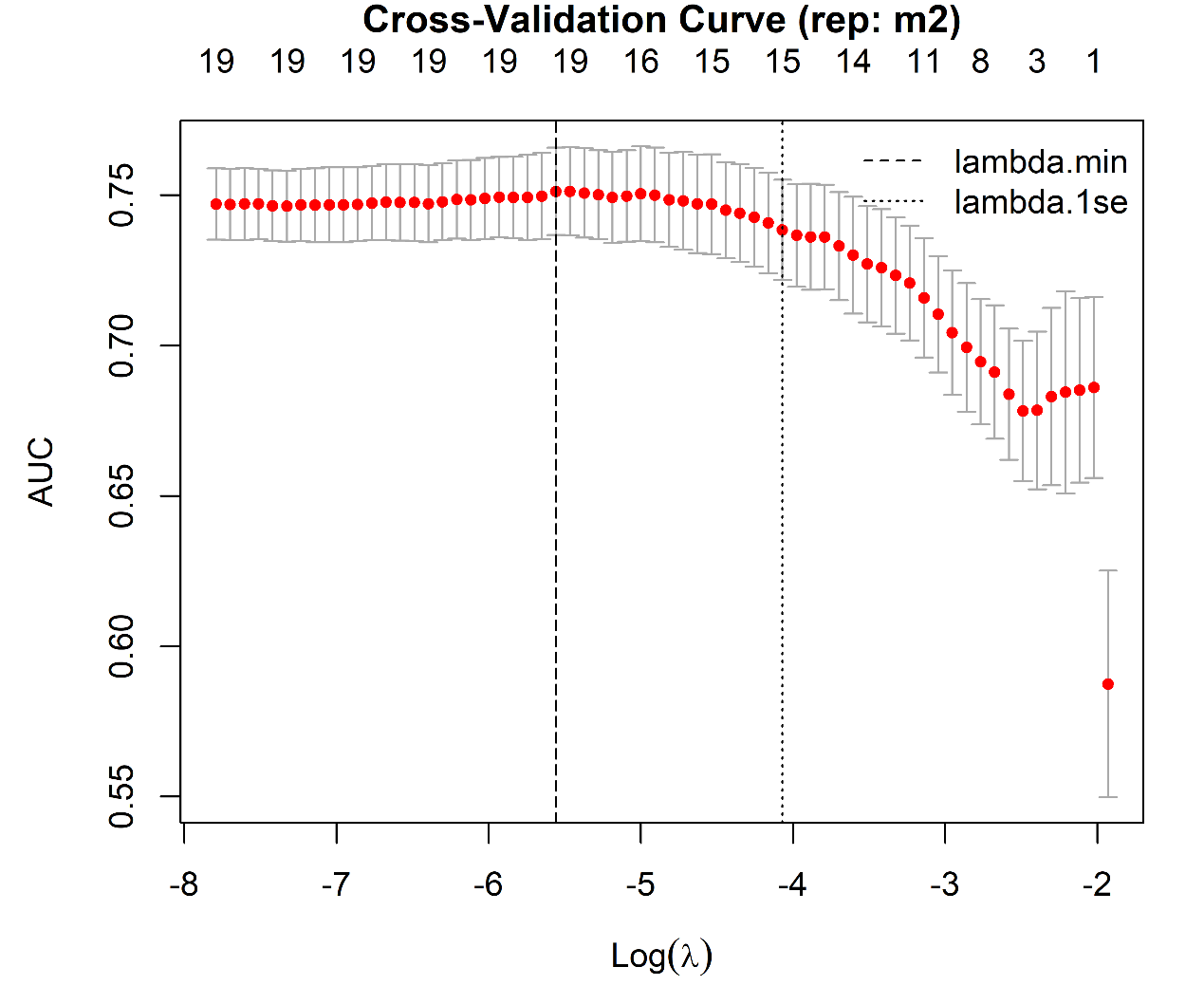


**Figure S2. Ten-fold cross-validation curve for the LASSO model (imputed dataset m2; sensitivity analysis).**The y-axis shows the mean AUC across folds; red dots denote fold-averaged AUC at each λ and error bars indicate ±1 standard error. Vertical dashed and dotted lines mark λ.min and λ.1se, respectively. Selecting λ.1se yields performance within 1 SE of the peak AUC while retaining fewer predictors, supporting a parsimonious model. Variable retention at λ.1se is summarized in Supplementary Table S2 and was directionally consistent with the primary multivariable analysis.

**Table S1. Multivariable logistic regression for odds of cardiovascular events (CVEs), with forced inclusion of age, diabetes, albumin, and B-type natriuretic peptide, in addition to the original predictors.**

| **Variable** | **OR** | **Lower 95% CI** | **Upper 95% CI** | **P-Value** |
| --- | --- | --- | --- | --- |
| Hypertension, n (%) | 3.23 | 1.54 | 6.75 | 0.002 |
| Muscle density, HU | 0.95 | 0.91 | 0.98 | 0.003 |
| Apolipoprotein B, g/L | 0.19 | 0.06 | 0.62 | 0.006 |
| Muscle volume, mm^3^ | 1.00 | 1.00 | 1.00 | 0.029 |
| Albumin, g/L | 0.95 | 0.91 | 1.00 | 0.038 |
| B-type natriuretic peptide, pg/mL | 1.00 | 1.00 | 1.00 | 0.051 |
| Age, years | 1.02 | 1.00 | 1.04 | 0.089 |
| Hemoglobin A1c, % | 1.12 | 0.88 | 1.41 | 0.357 |
| Diabetes, n (%) | 1.22 | 0.65 | 2.31 | 0.532 |

**Note:** Odds ratios (ORs) with 95% confidence intervals (CIs) are shown. Unit increments: muscle density per 1 HU; muscle volume per 1,000 mm³; albumin per 1 g/L; HbA1c per 1%; BNP per 100 pg/mL; age per 1 year; ApoB per 1 g/L. Hypertension and diabetes are Yes vs No (reference). Two-sided P values. Estimates are based on multiply imputed data (m = 5); this analysis serves as a sensitivity check and the direction of effects is consistent with the primary complete-case model.

**Table S2. Comparison of variable retention across modeling strategies.**

| **Variable** | **Original**  **multivariable logistic**  **regression** | **LASSO**  **(λ.1se, m=5)** | **Multivariable logistic regression**  **(with key covariates)** |
| --- | --- | --- | --- |
| Muscle volume, mm^3^ | Yes |  | Yes |
| Muscle density, HU | Yes | Yes | Yes |
| Hypertension | Yes | Yes | Yes |
| Apolipoprotein B, g/L | Yes |  | Yes |
| Hemoglobin A1c, % | Yes |  |  |
| B-type natriuretic peptide, pg/mL | Yes |  |  |
| Albumin, g/L |  | Yes | Yes |
| Parathyroid hormone, pg/mL |  | Yes |  |
| Serum creatinine, μmol/L |  | Yes |  |
| LDL-C, mmol/L |  | Yes |  |

**Note:** “Yes” indicates that the variable was retained at λ.1se in the LASSO model (based on m = 5 imputed datasets) and/or was retained in the specified multivariable logistic regression. Empty cells indicate non-retention. Variable names and units are aligned with the main manuscript: serum creatinine (μmol/L), BNP (pg/mL), muscle volume (mm³), muscle density (HU). This table summarizes selection/retention only; effect estimates are reported in the corresponding tables.
